# A Compact Dual-Band Dual-Mode Wearable Button Antenna for WBAN Applications

**DOI:** 10.3390/mi16090975

**Published:** 2025-08-25

**Authors:** Xue-Ping Li, Xue-Lin Zhang, Xue-Qing Yang, Zhen-Yong Dong, Xue-Mei Feng, Wei Li

**Affiliations:** 1 College of Electronic and Electrical Engineering, Henan Normal University, Xinxiang 453600, China; lixueping@htu.edu.cn (X.-P.L.); zhangxuelin202309@126.com (X.-L.Z.); 2322324016@stu.htu.edu.cn (X.-Q.Y.); 13938479502@163.com (Z.-Y.D.); fxm15037378353@163.com (X.-M.F.); 2 Henan Key Laboratory of Optoelectronic Sensing Integrated Application, Henan Normal University, Xinxiang 453600, China

**Keywords:** wireless body-area network (WBAN), dual-band dual-mode, button antennas, parasitic structure

## Abstract

A novel dual-band dual-mode wearable button antenna for wireless body area network (WBAN) applications is proposed in this paper. The antenna ingeniously integrates a monopole structure and an optimized planar inverted-F antenna (PIFA) configuration in a shared radiator, enabling dual-mode operation with a compact size. In the low-frequency band, the monopole structure generates an omnidirectional radiation pattern, facilitating efficient on-body communication. Meanwhile, the PIFA structure in the high-frequency band exhibits directed radiation, optimizing off-body communication. To enhance bandwidth, a parasitic structure is incorporated into the design. Both numerical simulations and experimental measurements are conducted to evaluate the antenna’s bandwidth and radiation performance in free space and on-body environments, with results showing excellent agreement. The measured bandwidth of the antenna on the human tissue is 300 MHz (2.3–2.6 GHz) in the low-frequency band and 4.5 GHz (5.5–10 GHz) in the high-frequency band. The maximum radiation efficiency reaches 76% in the low band (2.4–2.4835 GHz) and 93% in the upper band (5.725–5.875 GHz). Additionally, the peak gain on the human body can achieve 2.5 dB and 6.9 dB for the low and upper bands, respectively. The results confirm that the antenna meets the design requirements for Industrial, Scientific, and Medical (ISM) band applications, making it a promising candidate for WBAN systems.

## 1. Introduction

The advancement of wireless body area network (WBAN) technology necessitates the integration of radio-frequency (RF) antennas and circuits at a wide frequency band to enable efficient device-to-device communication [1,2,3,4,5,6]. Nowadays, WBANs are widely employed across various domains, including medical applications, health monitoring, and military operations [7,8,9]. With the emergence of flexible conductive materials, there has been growing interest in developing miniaturized and wearable electronics that ensure reliable wireless connectivity [10,11]. Thus, diverse wearable antenna designs have been explored.

Existing wearable antennas can be broadly classified into two categories. The first type is made of a flexible conductive material, which enables seamless integration with wearable systems. These antennas are designed using diverse approaches, including all-textile substrate [12], a thin flexible metal wire [13], and liquid metal-infused PDMS composites [14]. While their high conformability ensures comfortable wearability on the human body, the radiation performance can be degraded due to structural deformation caused by dynamic body movements. Furthermore, certain materials, such as liquid metal, are prone to inhomogeneous dispersion during fabrication or operation, potentially leading to unstable radiation characteristics and degraded efficiency. The second type of antenna is constructed from rigid materials, such as the frame of glasses in [15] or the structure of a watch in [16], which serve as the antenna substrate. Although these designs avoid deformation issues, they suffer from wearability challenges and limited applicability on the human body. Button antennas effectively address the limitations of both flexible and rigid antenna types. By integrating a rigid button head with flexible conductive textiles, this design prevents deformation while maintaining comfort during on-body wear, showcasing significant practical potential. The most common push-button antenna resembles the structure of the top hat monopole [17]. To accommodate diverse communication needs, a dual-band and dual-mode button antenna with an inverted top-load monopole structure and a planar inverted-F antenna (PIFA) structure is designed [18], but its higher frequency band remains relatively low. In [19], two different communication methods are realized using two PIFA structures, but the radiation performance is poor. Another design in [20] employs a hybrid top-loaded monopole and cross-dipole structure, enabling both linear and circular polarization across different frequency bands. However, this antenna’s structural complexity and high fabrication precision compromise its robustness.

In this work, a novel dual-band dual-mode button antenna that integrates monopole and PIFA structures to achieve two radiation performances in the impedance bandwidths is proposed. The antenna operates at 2.45 GHz with an omnidirectional monopole mode for efficient on-body communication while exhibiting directional radiation at 5.8 GHz for off-body communication. Through innovative structural design, the proposed antenna achieves significant miniaturization, making it the most compact solution among comparable designs. Furthermore, it delivers outstanding efficiency and gain across both operational bands, demonstrating superior performance for wearable applications.

## 2. Antenna Structure

**Figure 1:**
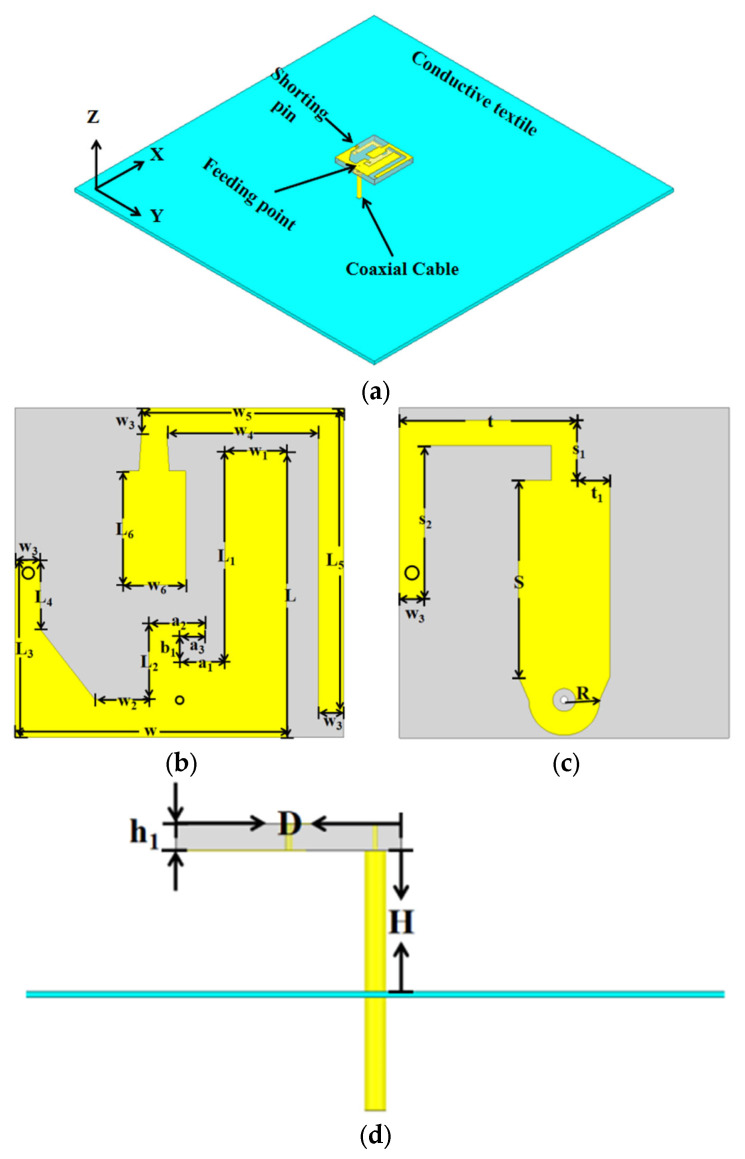
The antenna structure. (**a**) Overall top view; (**b**) Top view of the button; (**c**) Bottom view of the button; (**d**) Overall side view.

The proposed antenna design is illustrated in Figure 1 with detailed dimensional parameters as Table 1. It consists of a button head, a coaxial cable, and a metallized textile ground plane. The textile plane (side length of 100 mm, thickness of 0.1 mm) exhibits a high conductivity of 1.18 × 10^5^ S/m, ensuring effective performance in wearable applications. To obtain good and stable radiation performance, the button head employs an F_4_BM220 substrate (depicted in gray in Figure 1b) with patterned copper layers (shown in yellow in Figure 1b,c). The top layer incorporates a radiating element and parasitic structure, while the bottom layer features a ground plane, forming a compact, high-performance antenna configuration. Given the stringent size constraints of coaxial cables in button antenna design, the LA150 coaxial cable (Lair Microwave in the Suzhou, China) is selected to preserve the antenna’s impedance bandwidth and prevent high-frequency signal distortion. The LA150 coaxial cable serves as the center post whose inner conductor is connected to the top radiating element and outer conductor linked to the ground plane through an annular structure. The feed height H is designed to be approximately a quarter-wavelength at 5.8 GHz in free space. This height not only enables directional radiation at 5.8 GHz but also plays a critical role in tuning the low-frequency resonant characteristics.

## 3. Antenna Design Strategy and Radiation Principle

**Figure 2:**
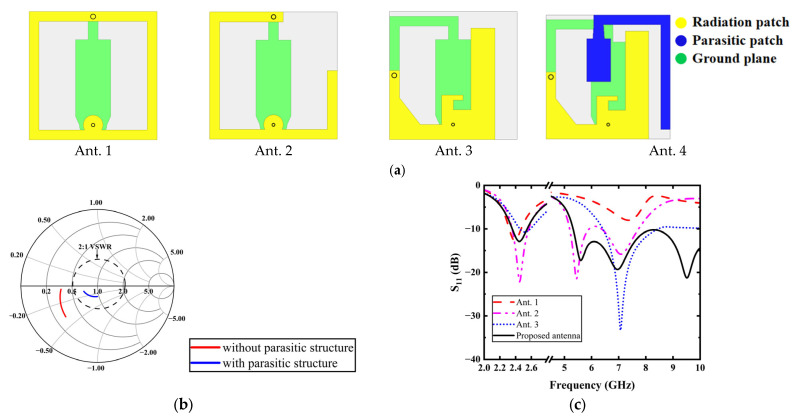
(**a**) Antenna evolution process; (**b**) Smith chart comparison of 5.8 GHz impedance with and without parasitic structure; (**c**) S_11_ with the antenna evolution.

The wearable button antenna design necessitates careful optimization of two critical characteristics: one is a miniaturized button head integrated with a large textile ground plane, and the other is a dual-mode radiating structure capable of supporting both on-body and off-body communication. Moreover, the antenna must demonstrate an omnidirectional radiation pattern in the low-frequency band for body-area networking, coupled with directional radiation in the high-frequency band for off-body links. To satisfy these requirements, the final antenna design incorporates a novel combination of an improved monopole and PIFA architectures, enabling both compact dimensions and dual radiation modes. The design evolution and impedance matching performance are comprehensively illustrated in Figure 2a,c.

To generate omnidirectional radiation at 2.45 GHz for on-body communications, the antenna structure integrates a top-loaded monopole configuration. The outer conductor of the coaxial is connected to a button head at one end and to a square-shaped plane made of flexible conductive textile at the other end. A ring radiator, etched on the button head’s substrate, is connected to the ground plane via a shorting pin, forming Ant. 1. The button head, acting as a capacitive load, is optimized to a side length of 13 mm to shift the resonance to 2.45 GHz. At this dimension, the loop structure introduces an additional resonance at 7 GHz, operating in loop mode. To create directional radiation at 5.8 GHz for off-body communications, the loop structure is reconfigured into an optimized PIFA to generate a new resonance at 5.5 GHz, as shown for Ant. 2. However, since this mode differs from the 7 GHz resonance, the −10 dB impedance bandwidth is limited to 700 MHz in the upper band, which is insufficient for multiple WBAN applications. Among various bandwidth enhancement techniques, the use of parasitic patches offers key advantages, including easy integration, design simplicity, and extremely low loss. To maintain the button’s compact form factor while preserving existing operational modes, Ant. 3 introduces structural modifications to the radiator and shorting-pin positioning to accommodate parasitic structure, albeit at the expense of degraded impedance bandwidth. To overcome this limitation, Ant. 4 presents the final optimized design, incorporating a parasitic structure to significantly enhance impedance bandwidth without sacrificing performance in either frequency band.

To visually assess the performance improvement, we compare the Smith chart characteristics at 5.8 GHz between two configurations of Ant. 3 (without parasitic structure) and Ant. 4 (optimized design incorporating parasitic structure). As shown in Figure 2b, the strategically positioned parasitic element adjacent to the L-shaped rectangular radiator achieves improved impedance matching through inductive coupling effects. Additionally, we can see after loading the parasitic structure in Figure 2b, the parasitic structure brings inductance for Ant. 3, which leads to the impedance curve of Ant. 3 moving into the VSWR = 2:1 circle on the Smith chart, significantly enhancing impedance matching in the high-frequency band. The final impedance bandwidth of the proposed antenna in the low-frequency band is broadened to 200 MHz (S_11_ < −10 dB), covering 2.35–2.55 GHz. Meanwhile, the high-frequency band can range from 5.3 GHz to 10 GHz. The antenna exhibits omnidirectional radiation in the low-frequency band and a directional radiation pattern in the high-frequency band, which meets the requirements of on-body communication and off-body communication.

**Figure 3:**
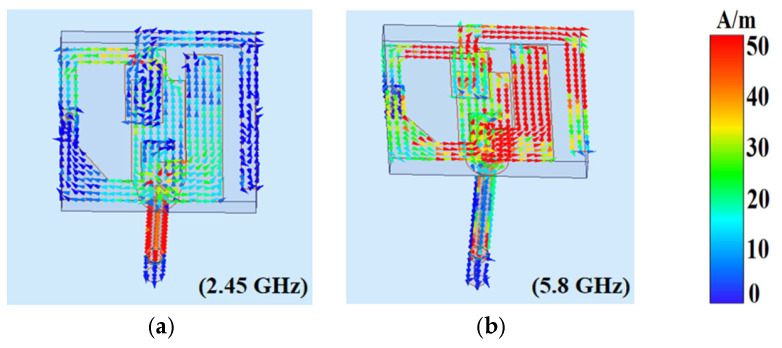
Surface current of button antenna at (**a**) 2.45 GHz; (**b**) 5.8 GHz.

To further demonstrate the working principle, Figure 3 shows the surface current of the button antenna at 2.45 GHz and 5.8 GHz. As depicted in Figure 3a, the surface current is predominantly concentrated on the loop section of the button head and the outer conductor of the coaxial cable. This configuration effectively establishes a top-loaded monopole structure, where the coaxial cable is the vertical monopole element and the loop section is the top-loaded element. This monopole arrangement facilitates omnidirectional radiation in the low-frequency band, rendering the antenna highly suitable for on-body surface communication applications.

Figure 3b shows the surface current of the antenna at 5.8 GHz. Most of the surface currents are distributed in the L-shaped stub, the parasitic structure, and the ground plane at the bottom of the substrate, confirming the antenna’s operational behavior at this frequency as well as the functional contribution of the parasitic element. In this configuration, the radiating structure connected by the shorting pin and the grounding plane constitutes an improved PIFA structure. The conductive textile serves as a reflector for high-band radiation, and its optimized positioning enhances directional radiation characteristics.

## 4. Antenna Robustness Test

To assess the robustness of the proposed button antenna in real-world wearable applications, we evaluate its performance under various mechanical deformations, including individual cases involving conductive textiles and coaxial cables, as well as scenarios where deformations are applied simultaneously. These common operational conditions are systematically modeled and analyzed to ensure reliable functionality in practical scenarios.

**Figure 4:**
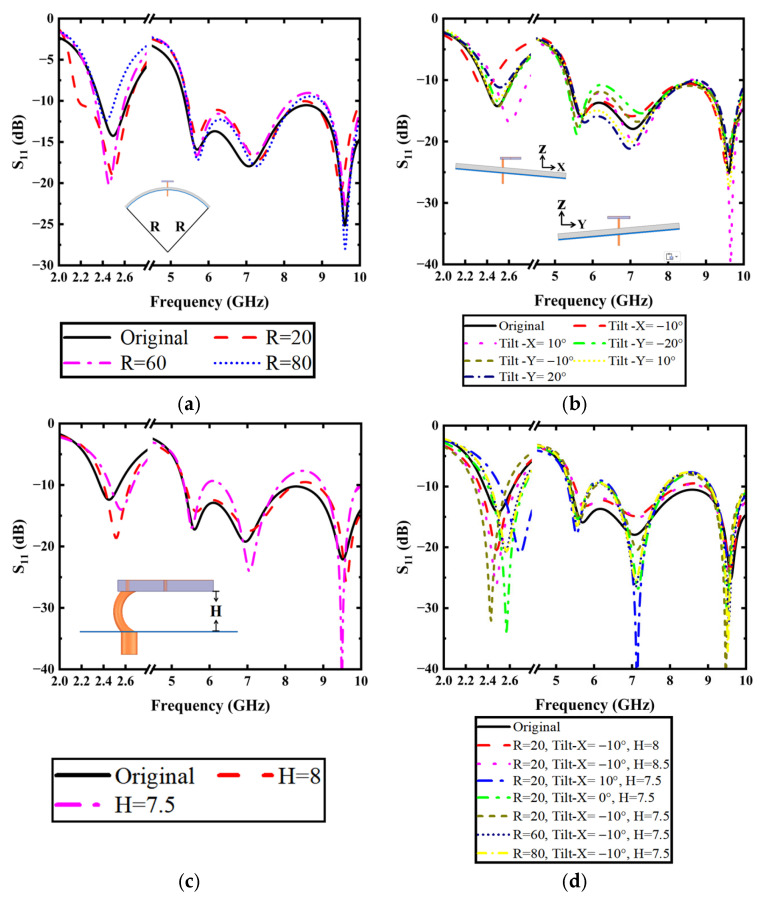
(**a**) The bending of conductive textiles with different radiuses of *R*; (**b**) Tilting the coaxial cable in different directions; (**c**) The bending of coaxial cable with different *H*; (**d**) Simultaneous bending of tilted conductive textiles and coaxial cable.

As shown in Figure 4a, the bending deformation is simulated by conforming the conductive textile to cylindrical surfaces with various radii (*R*), replicating the typical curvature encountered when worn on human limbs. Simulation results indicate that the antenna maintains excellent impedance matching (S_11_ < −10 dB) for bending radii exceeding 20 mm, validating its robustness for arm-worn applications. Figure 4b demonstrates the antenna’s performance under tilted conditions, where the conductive textile is tilted in the *x*-*z* plane and *y*-*z* plane. The results show that the maximum tilted angle at which the antenna’s bandwidth performance of both bands satisfies the design requirements from −20° to 20° in the *y*-*z* plane, while maintaining functionality within ±10° for tilting in the *x*-*z* plane.

To quantify coaxial cable bending, we employ the antenna’s profile height (*H*) as a proxy metric in Figure 4c. The results show that with the reduction in *H*, the low-frequency resonance shifts upward. The maximum allowable profile height for the antenna to sustain sufficient bandwidth performance in both frequency bands is 7.5 mm.

As illustrated in Figure 4d, the simultaneous bending of tilted conductive textiles and coaxial cable is simulated and analyzed. Given the numerous possible parameter combinations, our analysis focuses on several representative cases. In the following, we have selected the *x*-*z* plane inclination case as our representative example for analyzing tilted configurations. As shown in Figure 4d, under specific bending radius (*R*) and tilted angle (*X*) conditions of the conductive textiles, variations in profile height (*H*) produce opposing effects on the antenna’s low-frequency resonance compared to isolated changes in (*H*). When the bending radius (*R*) and profile height (H) remain constant, the antenna demonstrates increased sensitivity to tilted angle (*X*) variations, compromising its robustness in the *x*-*z* plane. In contrast, maintaining a fixed tilted angle (*X*) and profile height (*H*) leads to progressively stabilized impedance bandwidth performance as the bending radius (*R*) increases. In conclusion, the antenna’s impedance bandwidth demonstrates significant dependence on these parameter combinations, while the conductive textiles’ bending radius (*R*) exhibits relatively weaker influence on the overall performance characteristics. These comprehensive simulations confirm the antenna’s mechanical stability under realistic wearing conditions, demonstrating reliable performance despite the typical deformations encountered in wearable scenarios.

## 5. Simulation and Measurement Results

**Figure 5:**
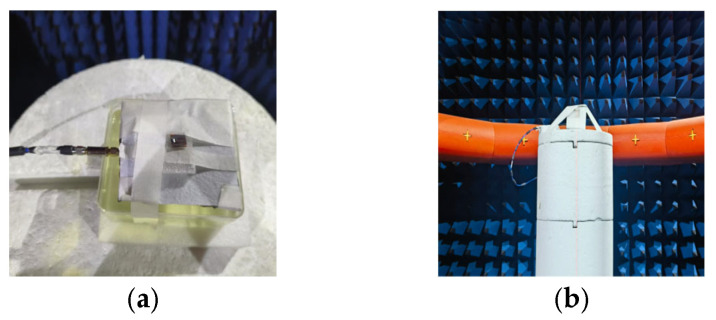
The actual fabricated antenna; (**a**) Top View; (**b**) Measurement setup in anechoic chamber.

Figure 5 presents photographs of the fabricated prototype of the proposed antenna. The design is experimentally characterized through both simulation and measurement to validate its performance. For comprehensive evaluation, two distinct test configurations are implemented: one is the fabricated antenna measured in free-space conditions, and the other is on-body scenario emulation with the antenna positioned 5 mm above tissue-mimicking liquid (prepared according to the formulation reported in [21]). This dual-test approach enables thorough assessment of the antenna’s performance in both isolated and body-proximity conditions.

**Figure 6:**
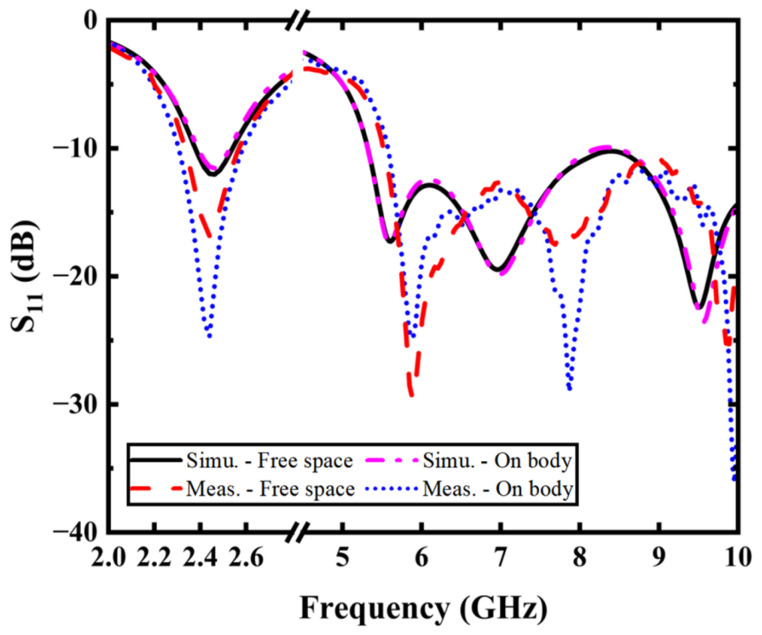
Measured and simulated S_11_.

The reflection coefficient results are shown in Figure 6. A good agreement to the simulation result is achieved for both free-space and on-body evaluations. For the measured reflection coefficient lower than −10 dB, the proposed antenna still covers the desired frequencies in two scenarios. The measured on-body impedance bandwidths are 300 MHz (2.3–2.6 GHz) in the low band and 4.5 GHz (5.5–10 GHz) in the high band. This thorough experimental validation confirms the design’s robust performance across both target frequency bands while accounting for practical deployment conditions in wearable applications.

**Figure 7:**
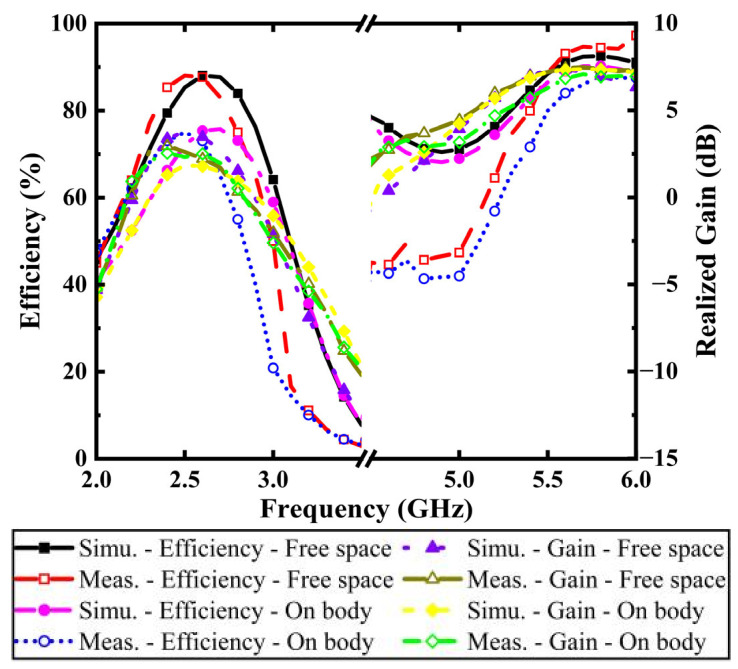
Measured and simulated efficiency and realized gain of the button antenna in free space and on body.

Figure 7 presents the simulated and measured radiation efficiency and realized gain of the proposed antenna in free space and on-body scenario. The results demonstrate that while the radiation efficiency and realized gain are decreased in the presence of a human body model, the design maintains remarkable efficiency levels of about 76% in the low band (2.4–2.4835 GHz) and 93% in the upper band (5.725–5.875 GHz). And the measured peak gains reaching 2.5 dB at 2.45 GHz and 6.9 dB in the 5.8 GHz.

**Figure 8:**
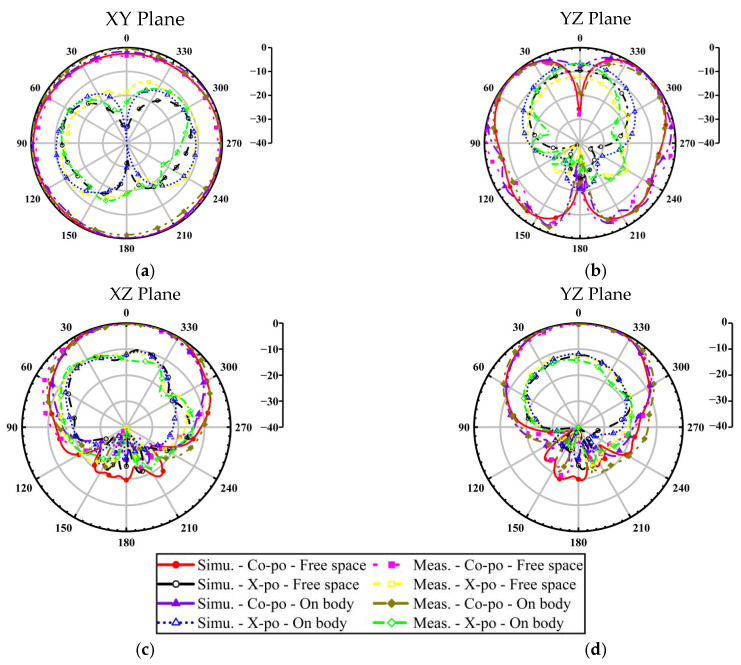
Measured and simulated normalized radiation patterns of the button antenna; (**a**,**b**) 2.45 GHz; (**c**,**d**) 5.8 GHz.

Figure 8 presents a comparative analysis of simulated and measured normalized radiation patterns, showing strong agreement between simulated and experimental results. Moreover, the button antenna presents an omnidirectional radiation pattern at 2.45 GHz for on-body communication scenarios, and a directional radiation pattern is presented at 5.8 GHz for off-body communication links. This dual-mode radiation behavior demonstrates the antenna’s versatility in wearable applications.

**Figure 9:**
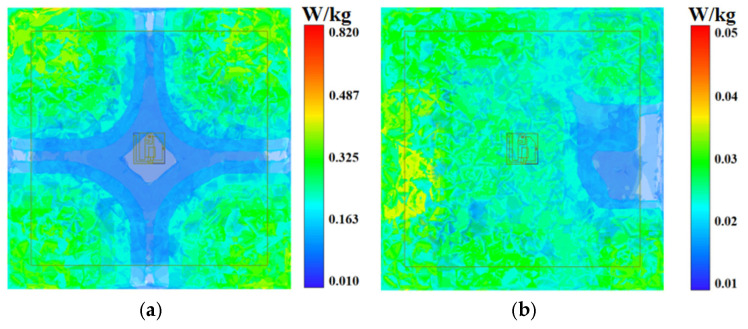
SAR distribution on a three-dimensional human body model. (**a**) 2.45 GHz; (**b**) 5.8 GHz.

To further assess electromagnetic safety compliance, we perform specific absorption rate (SAR) evaluations using a three-dimensional human tissue model (120 × 120 × 28 mm^3^). As shown in Figure 9, the maximum SAR values are 0.818 W/kg at 2.45 GHz and 0.047 W/kg at 5.8 GHz, well below the limit of 1.6 W/kg (1 g tissue). This stringent compliance ensures the design’s suitability for continuous, long-term wearable operation without health concerns.

The finalized antenna performance specifications are systematically compared with state-of-the-art designs in Table 2. Notably, the proposed antenna features the most compact button-head configuration among all compared implementations. In terms of bandwidth performance, the design achieves broader fractional bandwidths across both operational bands, outperforming the listed referenced works. Additionally, the design exhibits superior gain characteristics compared with other designs except for [20].

## 6. Conclusions

To address the stringent demands of wearable communication systems, this work presents a novel compact dual-band dual-mode button antenna with an innovative hybrid architecture. The design synergizes a top-loaded monopole radiating structure with an optimized PIFA configuration, further enhanced by a strategically integrated parasitic element to broaden operational bandwidth. The antenna efficiently supports dual-band operation at the ISM bands of 2.45 GHz and 5.8 GHz. Both simulation and experimental results demonstrate that the proposed antenna exhibits excellent dual-band performance with satisfactory bandwidth characteristics. In the low-frequency band, the antenna operates in monopole mode, providing an omnidirectional radiation pattern ideal for on-body communication. Meanwhile, in the upper band, it generates a wide-side radiation pattern, optimized for efficient off-body signal transmission. The antenna achieves outstanding radiation efficiency of 76% at 2.45 GHz (2.4–2.4835 GHz) and 93% at 5.8 GHz (5.725–5.875 GHz), and the peak gain on the human body can achieve 2.5 dB and 6.9 dB, respectively. All the results conclusively demonstrate that the proposed antenna possesses superior performance characteristics, positioning it as an ideal candidate for emerging wearable communication applications.

## Figures and Tables

**Table 1:** Antenna parameters.

Parameters	Value (mm)	Parameters	Value (mm)
L	11.3	w_4_	6
L_1_	8.3	w_5_	8
L_2_	3	w_6_	2.5
L_3_	7	S	7.75
L_4_	2.75	s_1_	2.35
L_5_	12	s_2_	6
L_6_	4.5	t	7
w	10.75	t_1_	5
w_1_	2.45	a_1_	1.8
w_2_	2.15	a_2_	2.2
w_3_	1	a_3_	1
D	13	b_1_	1
H	8.5	h_1_	1.5
R	1.4		

**Table 2:** Comparison table of typical button antennas.

Ref.	Area (mm × mm)	Size Comparison	Profile (mm)	f_0_ (GHz)	−10 dB|S_11_| BW (GHz)	Radiation Pattern	Antenna Type	Gain (dBi)
[18]	π × 100	186%	9	2.45 5.80	2.33–2.62 (12%) 5.13–8.03 (44%)	O&D	Monopole PIFA	1.2/5.7
[19]	π × 81	151%	4	2.45 5.80	2.4–2.5 (4%) 5.70–5.90 (3%)	O&D	PIFA PIFA	−0.6/4.3
[20]	π × 81	151%	10	2.45 5.80	2.39–2.57 (7%) 4.80–8.18 (52%)	O&D	Monopole Cross-Dipoles	2.2/8.6
[22]	π × 95	177%	8	5.50 5.70	5.43–6.11 (12%)	D	N/A	3.5
[23]	π × 64	119%	6	2.45 5.80	2.36–2.49 (6%) 4.66–6.9 (39%)	O&D	Monopole Patch	0.24/4.29
This work	13 × 13	100%	8.5	2.45 5.80	2.3–2.6 (12%) 5.50–10.0 (58%)	O&D	Monopole PIFA	2.5/6.9

## Data Availability

The original contributions presented in this study are included in the article. Further inquiries can be directed to the corresponding author.
